# Drug-Induced Liver Injury Caused by Amoxicillin/Clavulanate

**DOI:** 10.7759/cureus.12234

**Published:** 2020-12-23

**Authors:** Inês Ferreira, Cláudio Gouveia, Carolina Vasques, Catarina Faria, Ana Pedroso

**Affiliations:** 1 Internal Medicine, Hospital São Francisco Xavier, Lisboa, PRT

**Keywords:** drug, liver, injury, amoxicillin/clavulanate, jaundice

## Abstract

Drug-induced liver injury (DILI) has a relatively low incidence, and as it is a diagnosis of exclusion, it can become quite a challenge for the clinician. Amoxicillin/clavulanate continues to be one of the most prescribed antibiotics and only rarely causes liver injury. We report a case of DILI associated with this antibiotic to bring attention to a rare side effect of a very commonly prescribed drug. This is the case of a 71-year-old man, with no relevant past medical history, who presented to the Emergency Department due to jaundice in the previous two weeks, with no immediate identifiable cause. The patient was admitted to our Internal Medicine Ward, and after getting a detailed clinical history and excluding other common and severe causes of liver injury, the diagnosis was made that liver injury was due to amoxicillin/clavulanate intake, thus demonstrating the importance of an in-depth history.

## Introduction

Drug-induced liver injury (DILI) is a rare entity (estimated annual incidence between 10 and 15 per 100,000 persons exposed to prescription medications) [1], which can be classified as hepatocellular, cholestatic, or mixed. Cholestatic DILI is defined as an elevation of alkaline phosphatase greater than twice the normal level and/or an alanine aminotransferase/alkaline phosphatase ratio less than 2. Mixed DILI presents with an alanine aminotransferase/alkaline phosphatase ratio that is greater than 2 but less than 5, being an intermediate between cholestatic and hepatocellular liver injury [2]. Cholestatic DILI is the most common type in the elderly and takes the longest to resolve after drug suspension. Most cases are mild; however, approximately 10% of the cases can progress to cirrhosis [2,3]. DILI is a diagnosis of exclusion, resulting in a challenge for the clinician [4].

The traditional diagnostic approach of DILI involves a clinical, laboratorial, and sometimes histological evaluation. It is necessary to establish a time link between drug exposure and the establishment of liver injury, exclude other causes of liver injury, confirm resolution of liver injury after drug suspension, and, in some cases, evaluate the effect of re-exposure. DILI diagnosis can be complex due to the inherent subjectivity of this approach; to solve this problem, diagnostic strategy tools such as the Roussel-Uclaf Causality Assessment Method (RUCAM) were developed [4].

Amoxicillin is a semi-synthetic antibiotic that was combined with clavulanate to prevent degradation by enzymes such as beta-lactamase. Commercialized since 1981, it continues to be one of the most prescribed antibiotics to treat a wide variety of infections. According to a report published by the Centers for Disease Control and Prevention, there were 26.6 million prescriptions of amoxicillin/clavulanate made in 2018, making it the third most prescribed antibiotic in the United States [5].

This case reports an example of jaundice secondary to the use of amoxicillin/clavulanate that was prescribed to treat a respiratory infection. Our aim is to highlight a rare side effect of a very commonly used drug and the associated risk factors. We also wish to draw attention to the fact that the clinical presentation can be quite acute and can occur even after the patient has suspended the antibiotic, which can mislead the clinician into considering obstruction or neoplasms as the most probable diagnosis during the differential diagnosis. Only a complete and in-depth history can avoid unnecessary and invasive examinations.

## Case presentation

We report the case of a 71-year-old male, with no medical history and no chronic medication. He presented to the Emergency Department complaining of jaundice for two weeks associated with acholia, choluria, pruritus, and a mild rash involving the limbs and torso. He denied having fever, headache, myalgias, arthralgias, respiratory symptoms, abdominal pain, nausea, vomiting, diarrhea, genitourinary symptoms, anorexia, or weight loss. The patient had no known allergies, and there was no exposure to alcohol, tobacco, or any illicit drugs. No relevant family history findings were present.

On physical examination, the patient presented with no fever and hemodynamic stability. The skin and sclera showed visible jaundice, and a generalized, mild, macular, non-petechial rash was also present. Abdominal examination showed a completely normal, painless abdomen, without hepatosplenomegaly or palpable masses, without any signs of chronic liver disease or portal hypertension. Neurological examination was completely normal. Multiple medical tests were performed to determine the cause of jaundice. Laboratory analysis (Table 1) revealed a cholestatic pattern with hyperbilirubinemia caused by elevated levels of conjugated bilirubin. Abdominal ultrasound and computerized tomography (CT) scan showed a liver with normal dimensions, with no structural anomalies or lesions, a gallbladder with no evidence of gallstones, and bile ducts with maintained caliber. There were no relevant findings concerning the pancreas.

**Table 1:** Blood and urine laboratorial results in the Emergency Department.

	Relevant Laboratorial Results	Reference Values
Hemoglobin	13.6 g/dL	13.0-17.0 g/dL
Mean Corpuscular Volume	86.1 fL	80.0-96.1 fL
Mean Corpuscular Hemoglobin	28.0 pg	27.3-33.7 pg
Leucocytes	6.2 x 10^9^/L (0% eosinophils)	4.0-10.0 x 10^9^/L
Platelets	306 x 10^9^/L	150-400 x 10^9^/L
Prothrombin Time	11.6 seconds	<14 seconds
Urea	27 mg/dL	17-49 mg/dL
Creatinine	0.76 mg/dL	0.7-1.20 mg/dL
Sodium	140 mmol/L	136-145 mmol/L
Potassium	4.66 mmol/L	3.5-5.10 mmol/L
Albumin	4.1 g/dL	3.5-5.2 g/dL
Total Bilirubin	8.79 mg/dL	<1.40 mg/dL
Conjugated Bilirubin	6.21 mg/dL	<0.3 mg/dL
Alanine Transaminase	193 U/L	<41 U/L
Aspartate Transaminase	69 U/L	<40 U/L
Gamma-Glutamyltransferase	190 U/L	10-71 U/L
Alkaline Phosphatase	262 U/L	40-130 U/L
Lactate Dehydrogenase	262 U/L	135-225 U/L
Lipase	19 U/L	13-60 U/L
Thyroid-Stimulating Hormone	1.0 uUI/mL	0.27-4.20 uUI/mL
C-Reactive Protein	1.75 mg/dL	<0.5 mg/dL
Urinalysis: Absence of proteins, glucose, ketone bodies, urobilinogen, and hemoglobin. Bilirubin (+).		
Serology panel: Hepatitis A virus immunoglobulin M, hepatitis B surface antigen, and hepatitis C virus antibodies were negative. Anti-HIV antibodies were also negative.		

Due to the need for further investigation, the patient was admitted to the Internal Medicine Ward. A more detailed clinical history acquired during the hospital stay revealed that the patient had been taking amoxicillin/clavulanate 875/125 mg twice a day for a respiratory infection for 14 days. Two days after completing the antibiotic scheme, the patient presented with mild jaundice of the scleral conjunctiva (16 days after treatment with amoxicillin/clavulanate was initiated), which progressed over the next two weeks, motivating the patient to seek medical help in the Emergency Department.

Even though there was a temporal link between the drug and the development of liver injury, DILI is a diagnosis of exclusion, and due to the patient’s age and the exuberance of the analytical findings, other causes of cholestasis had to be excluded. The differential diagnosis of hyperbilirubinemia due to elevated conjugated bilirubin must take into account intra- and extrahepatic cholestasis. For this reason, during the hospital stay, the patient was submitted to various tests, including immunological investigation (Table 2), iron studies (Table 3), and additional serology (Table 4).

**Table 2:** Immunology panel with no relevant findings, thus eliminating an autoimmune cause for the liver injury. IgG, immunoglobulin G

	Results	Reference Values
Rheumatoid Factor (U/mL)	10	<15
Antinuclear Antibodies	Negative	
Extractable Nuclear Antigen Antibodies	Negative	
Anti-Mitochondrial Antibodies	Negative	
Anti-Smooth Muscle Antibodies	Negative	
Anti-Soluble Liver Antigen Antibodies	Negative	
Anti-Neutrophil Cytoplasmic Antibodies	Negative	
IgG (mg/dL)	1640	600-1500
IgG Subclass 1 (mg/dL)	977	422-1290
IgG Subclass 2 (mg/dL)	883	117-747
IgG Subclass 3 (mg/dL)	151	40-130
IgG Subclass 4 (mg/dL)	25	1-291
Immunoglobulin M (mg/dL)	144	50-300
Immunoglobulin A (mg/dL)	610	50-400

**Table 3:** Iron studies with no relevant alterations.

Iron Studies	Results	Reference Values
Iron (ug/dL)	70	33-193
Ferritin (ng/mL)	206	18-464
Total Iron-Binding Capacity (ug/dL)	261	250-425
Transferrin Saturation (%)	27	20-55

**Table 4:** Continuation of serological investigation that was started in the Emergency Department and was completed during the hospital stay. IgG, immunoglobin G; IgM, immunoglobulin M

Virus	IgG	IgM
Cytomegalovirus	Negative	Negative
Epstein-Barr	Positive	Negative
Varicella Zoster	Negative	Negative
Herpes Simplex Type 1/2	Negative	Negative
Hepatitis E	Negative	Negative

There was no sign of infection, neoplasm, and autoimmune or metabolic disease either clinically, biochemically, or radiologically (serological panel was negative for hepatitis A, B, C, E, cytomegalovirus, Epstein-Barr virus, herpes simplex virus, and varicella zoster virus; serological levels of IgG4 were normal; antibodies for primary biliary cholangitis were negative; thyroid function was normal; iron studies were normal). Through abdominal imaging with magnetic resonance cholangiopancreatography, we were also able to confirm that the liver, gallbladder, and pancreas had conserved dimensions, with no identifiable nodular lesions, and eliminate the possibility of intra- or extrahepatic obstruction. In light of the patient's age, absence of family history, and lack of clinical, biochemical, and radiological support, the diagnosis of hereditary and infiltrative diseases was not pursued, and hence the decision to not perform liver biopsy.

The patient was discharged at the seventh day of admission. No specific treatment was given apart from fluid therapy and occasionally antihistaminic drugs for pruritus.

**Figure 1:**
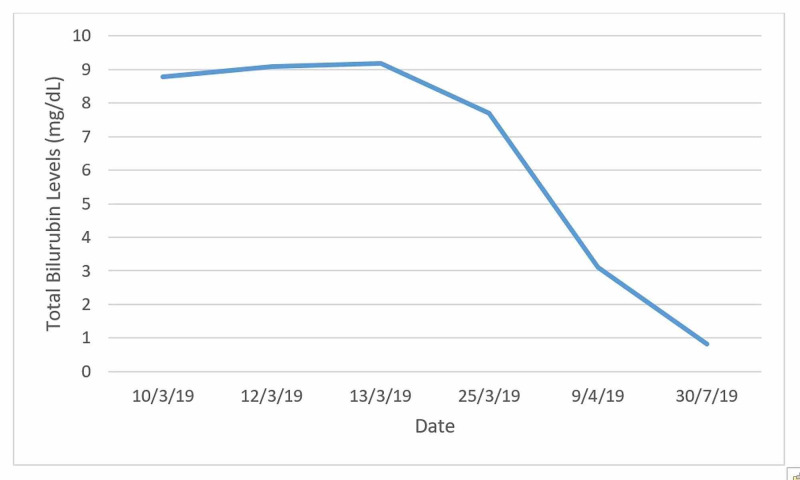
Five-month evolution of total bilirubin levels until normalization.

**Figure 2:**
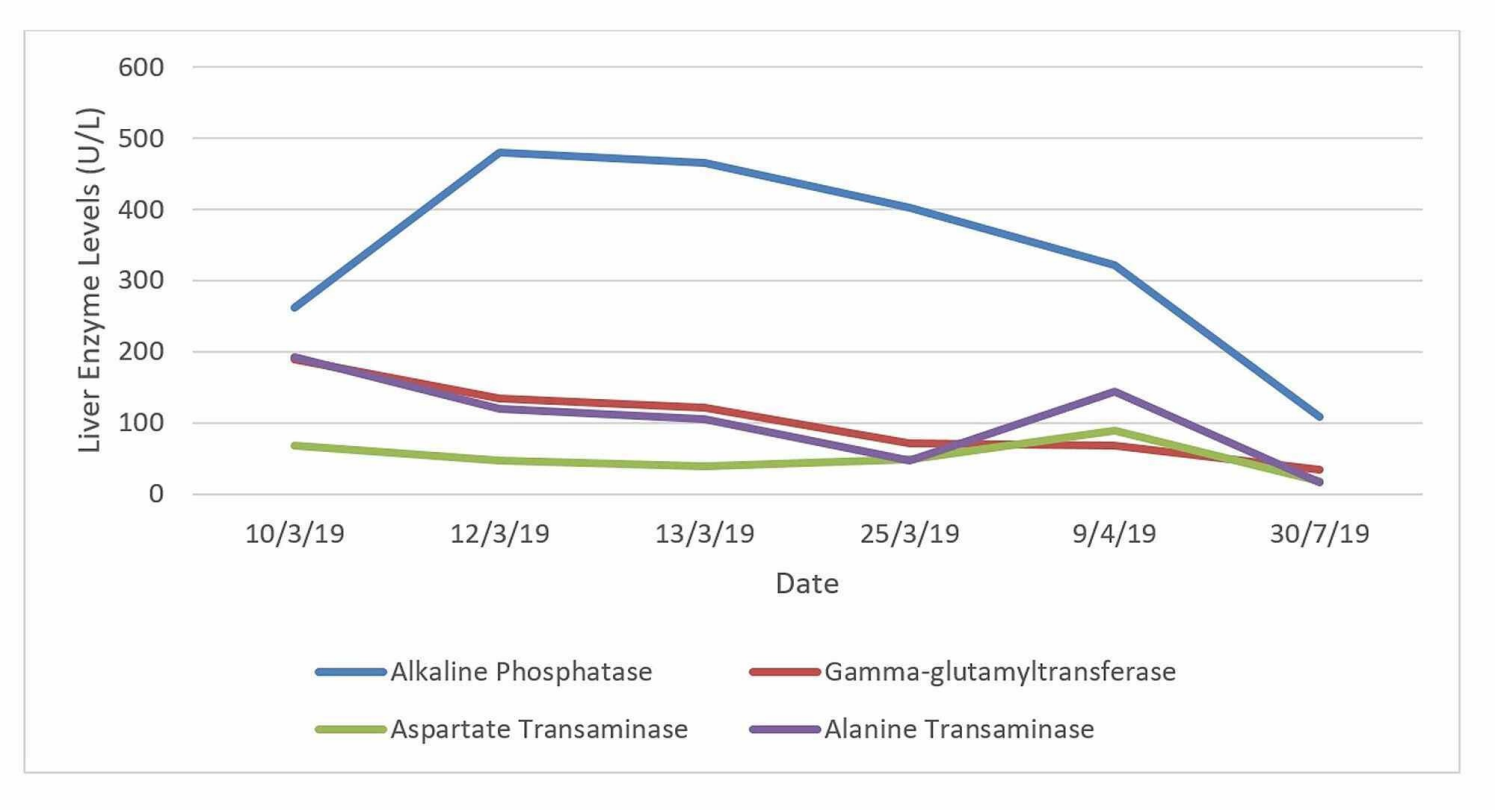
Five-month evolution of liver enzymes until normalization.

Through the patient’s follow-up we were able to confirm the resolution of all the findings previously mentioned after a five-month period (<180 days) since antibiotic suspension (Figures 1, 2). Using the RUCAM scale our case scored nine points, making the DILI diagnosis very probable [6].

## Discussion

We believe that it is important to report this case due to the fact that amoxicillin/clavulanate is a very frequent and widely used antibiotic [7]. The incidence of amoxicillin/clavulanate-induced liver injury is greater than that induced by amoxicillin alone, and although it can cause any type of DILI, cholestasis is the most frequent kind of presentation [7,8].

The pathogenesis behind amoxicillin/clavulanate liver injury requires more studies; however, it is believed that immuno-allergic mechanisms are involved. The presence of eosinophils in the inflammatory infiltrate, hypersensitivity manifestations such as skin rashes and hyper-eosinophilia, and the association with human leucocyte antigen (HLA) class II genes (DRB1 * 1501-DRB5 * 0101-DQB1 * 0602) reinforce the hypothesis that an immune aggression is involved in the liver injury caused by this antibiotic [7-9]. Risk factors include male sex, alcohol consumption, repeated use of the drug, concomitant use of other hepatotoxic drugs, and age over 55 years [7,8,10]. The duration of treatment was also included as a risk factor in some reviews [7].

Clinical manifestations are predominantly cholestatic, namely anorexia, nausea, vomiting, jaundice, choluria, acholia, pruritus, and, occasionally, painful hepatomegaly. Hypersensitivity manifestations, such as skin rash and fever, can sometimes occur [7-9]. DILI can be considered up to 90 days after initial drug intake or up to 30 days after drug suspension. This clinical condition can take up to six months after drug suspension to completely resolve [6]. Even though most cases have a benign course, on rare occasions it can progress to liver failure, requiring close monitoring [7,8]. Treatment consists of withdrawal of the offending drug and supportive treatment such as fluid replacement. Cholestatic symptoms can become very limiting and demand treatment with antiemetic and analgesic drugs, as well as cholestyramine, antihistamine, ursodeoxycholic acid, or even sertraline to control pruritus, depending on its severity. Due to the fact that an immune mechanism might be involved in DILI, corticotherapy has been suggested, but there are no supporting data that suggest a reduction in morbidity [11]. 

This case describes a 71-year-old man who started to manifest signs of cholestatic jaundice two days after suspending treatment with amoxicillin/clavulanate after a 14-day treatment. The patient developed acute conjugated hyperbilirubinemia with a cholestatic pattern of the liver function tests, however with no other severity criteria of impending severity. The complete clinical and biochemical resolution lasted five months, with no specific treatment. Several risk factors for DILI were present in this case, namely, advanced age, male sex, and prolonged exposure to the drug. A complete anamnesis and extensive biochemical and radiological examinations excluded other common causes of liver injury. RUCAM score was applied (nine points), making DILI highly probable. A relevant factor, which complicated the diagnosis, was that the patient denied recent intake of drugs when initially interviewed. Liver biopsy was not performed since other common causes of liver injury were excluded, making DILI secondary to amoxicillin/clavulanate the most probable diagnosis. When DILI is the most probable diagnosis and other causes of liver injury have been eliminated, the best course of action is vigilance and monitoring, certifying a decrease in liver enzyme levels, and thus sparing the patient from painful and invasive procedures. Exposure to amoxicillin/clavulanate should be avoided in the future [7,8].

## Conclusions

This clinical report demonstrates the importance of a complete and detailed patient history and a responsible use of antibiotics. This is especially important in at-risk groups, a population that is a frequent user of health services and is more susceptible to infection.

As amoxicillin/clavulanate is a very commonly used antibiotic, reports such as this one are fundamental to highlight this rare side effect so that medical professionals become more aware of its existence. By doing so, we hope to spare patients of unnecessary examinations, which can be invasive and with potential serious complications.
